# Advanced Cognitive Impairment Outpatient Clinic and the importance of multidisciplinary care in the care of patients with advanced dementia

**DOI:** 10.1002/alz.093218

**Published:** 2025-01-09

**Authors:** Carolina Saad Hassem, Ana Carolina Borges de Miranda Souza, Rafael Tobias Athias, Natália Mira Gonçalves, João Otavio Sá dos Reis, Wilson Jacob‐Filho, Lilian Schafirovits Morillo

**Affiliations:** ^1^ Hospital das Clínicas da Faculdade de Medicina da Universidade de São Paulo, São Paulo, São Paulo Brazil; ^2^ Hospital das Clínicas da Faculdade de Medicina da Universaidade de São Paulo, São Paulo, São Paulo Brazil; ^3^ University of São Paulo Medical School, São Paulo, São Paulo Brazil

## Abstract

**Background:**

Dementia is characterized by a chronic and progressive loss of cognitive function in the absence of a fluctuating level of consciousness. Alzheimer’s disease is the main etiology of dementia, presenting insidiously and causing progressive cognitive impairment with increasing severity over the years. Given the immense complexity of managing patients with advanced dementia, the Advanced Cognitive Impairment Outpatient Clinic (ACIOC) of the Geriatric Service of the Internal Medicine Division of the Hospital das Clínicas of the Faculty of Medicine of the University of São Paulo (HCFMUSP) was created in April 2008, which aimes to monitor elderly patients referred from other outpatient clinics, with moderate to advanced dementia, for multidisciplinary follow‐up: doctor, speech therapist, occupational therapist and dentist. Given the complexity of patients with advanced dementia, each patient is assessed, in person, on average, 4 times a year.

**Method:**

To describe the functioning and sample of patients that make up the ACIOC, according to data collected via electronic medical records between August 2018 and January 2024.

**Result:**

Electronic medical records were implemented in August 2018. Data taken from records indicate that 246 patients were assisted between August 2018 and January 2024, with an average age of 79.10 years. The majority of patients were white (60%), female (65%) and had 5 years of schooling, on average. The main comorbidities were: hypertension (40%), coronary artery disease (40%) and diabetes mellitus (17%). Regarding the etiology of dementia, Alzheimer’s was the most prevalent (28%), followed by Vascular (15%), Mixed (12%), Frontotemporal (5%), LBD (4,8%) and 23% of patients did not have diagnosis. Regarding the treatment carried out, 61% were treated with anticholinesterases, 59% with antidepressants, 53% with antipsychotics and 46% with memantine.

**Conclusion:**

The data obtained highlights the importance of ACIOC in dementia care, especially for those residing in São Paulo / Brazil. This study seeks not only to contribute to the understanding of dementia in the Brazilian context, but also emphasizes the need to share these findings to improve services offered in other institutions dedicated to the care of these patients.

